# Evaluation of New and Preexisting Epiretinal Membranes Following Glaucoma Filtration Surgery

**DOI:** 10.7759/cureus.46441

**Published:** 2023-10-03

**Authors:** Ryota Aoki, Saki Dote, Satomi Oogi, Yuki Nagata, Kanae Ueda, Etsuko Terao, Shunsuke Nakakura

**Affiliations:** 1 Ophthalmology, Saneikai Tsukazaki Hospital, Himeji, JPN

**Keywords:** ex-press, trabeculectomy, secondary epiretinal membranes, filtration surgery, glaucoma

## Abstract

Secondary epiretinal membranes (ERMs) can develop from various causes, including those associated with glaucoma treatments such as trabeculectomy (TLE) and EX-PRESS (EXP) insertion surgery. This study aimed to investigate the occurrence of new ERMs and changes in preexisting ERMs following TLE or EXP insertion. Between April 2018 and March 2019, 102 and 74 eyes that underwent primary and standalone TLE and EXP insertion, respectively, were evaluated. Of these, 48 eyes were included in the TLE group and 32 eyes were included in the EXP group. Optical coherence tomography (OCT) was used to assess preoperative and postoperative ERMs. In the TLE group, postoperative ERMs were observed in one (case 1) (3%) out of 34 eyes without preexisting ERMs and in one (case 2) (7%) out of 14 eyes with preexisting ERMs, showing an increase in ERM stage. In the EXP group, postoperative ERMs were observed in one (case 3) (5%) out of 22 eyes without preexisting ERMs and in one (case 4) (10%) out of 10 eyes with preexisting ERMs, showing a decrease in the ERM stage. Case 1 was a 58-year-old man with primary open-angle glaucoma (POAG) in the left eye who underwent TLE. Although no preoperative ERMs were observed, postoperative ERM was noted at the three-month follow-up. Case 2 was a 49-year-old man with POAG in the right eye who underwent TLE. Although ERM was observed preoperatively, ERM progressed at six months postoperatively. Case 3 was a 59-year-old woman with POAG in the right eye who underwent EXP insertion. No preoperative ERMs were observed, but an ERM was noted at the 15-month follow-up. Case 4 was a 72-year-old woman with steroid-induced glaucoma in the right eye who underwent EXP insertion surgery. A preoperative ERM was present, and the foveal pit was absent; however, the foveal pit was observed at the 12-month follow-up. Despite the low incidence of ERMs, filtration surgery may be associated with ERM development and the progression or regression of preexisting ERMs.

## Introduction

Epiretinal membranes (ERMs) are mostly idiopathic [1], with advanced age being its most significant risk factor [2-4]. A meta-analysis involving 13 studies reported an overall prevalence rate of 9.1% for ERMs [2]. Furthermore, the incidence of new ERMs during a five-year follow-up was 5.3% [5]. Secondary ERMs can develop from various causes, including retinal vascular diseases [6], uveitis [7], retinal tears, rhegmatogenous retinal detachment [1,8,9], pathological myopia, trauma, intraocular tumors, and age-related macular degeneration [1]. Among those associated with glaucoma, ERM development has been reported after trabeculectomy (TLE) [10] and EX-PRESS (EXP) insertion surgery [11]. In this study, we investigated the incidence of new ERMs and the status of preexisting ERMs following TLE or EXP insertion.

## Materials and methods

**Figure 1:**
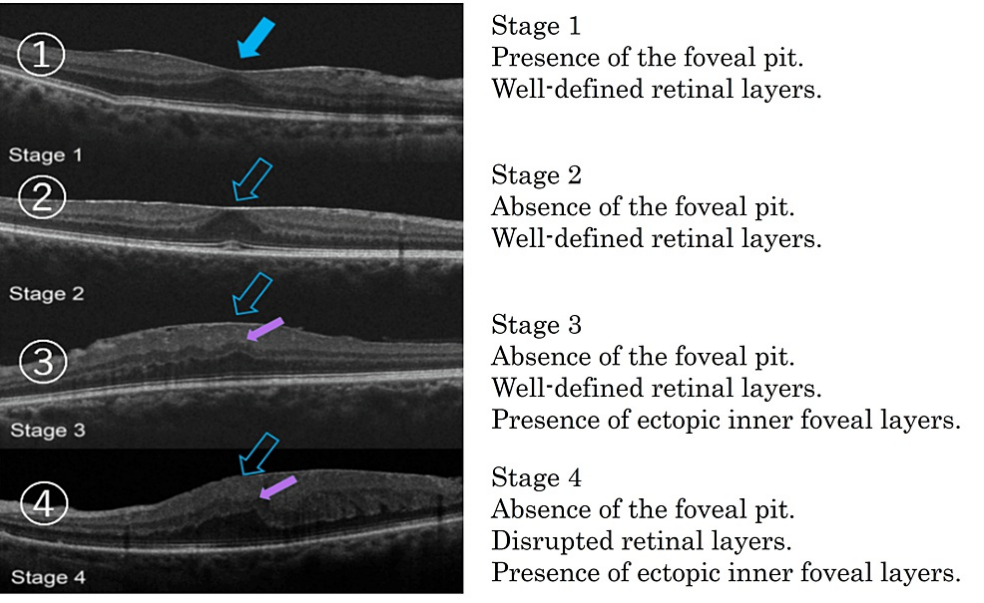
Epiretinal membrane staging by Govetto et al. A figure was constructed based on the findings reported by Govetto et al. [12].

This study was approved by the Institutional Review Board of Saneikai Tsukazaki Hospital (IRB no. 231004) and was conducted according to the tenets of the Declaration of Helsinki. We conducted a retrospective analysis of patients who underwent primary and standalone TLE (102 eyes) or EXP insertion surgery (74 eyes) performed by a single surgeon at Tsukazaki Hospital between April 2018 and March 2019. Inclusion criteria were as follows: 1) a postoperative follow-up period of at least one year; 2) availability of optical coherence tomography (OCT) (3D OCT-2000, Topcon Corp., Japan) imaging data before and after surgery on at least two occasions (the device utilized in the past at Tsukazaki Hospital lacks its original data and suffers from inconsistent color calibration); 3) no history of other glaucoma surgery, excluding needling, and filtration bleb revision, within two years after TLE or EXP insertion surgery; and 4) no history of other intraocular surgery within one year prior to TLE or EXP insertion surgery. OCT images were captured at approximately three-month intervals. ERM staging was performed using the classification proposed by Govetto et al. [12] (Figure 1). OCT imaging was utilized to evaluate ERMs.

Surgical techniques

The TLE procedure was performed via a fornix-based conjunctival dissection. Initially, a 7 mm conjunctival incision was made along the corneal limbus, followed by sub-Tenon's anesthesia using 2% lidocaine hydrochloride. The sclera was exposed, and a 2.5 mm × 2.5 mm square half-thickness scleral flap was created. A 0.04% Mitomycin C was applied for three minutes and then washed out with 50 mL of saline solution. Subsequently, a 2 mm × 2 mm second flap was created and excised. After peripheral iridectomy, the scleral flap was sutured with 10-0 nylon sutures. Finally, the conjunctiva was sutured at the limbus using 10-0 nylon sutures. The EXP insertion procedure was similar to the TLE procedure until the application of Mitomycin C. After Mitomycin C was washed out, the EXP device was inserted, and the scleral flap was sutured with 10-0 nylon sutures. Conjunctival closure was performed in a manner similar to that of the TLE procedure. After surgery, all cases received topical 1.5% levofloxacin and 0.1% betamethasone four times daily for one month and tapered over a period of one to three months, respectively. 

## Results

This study included 48 and 32 eyes that underwent TLE and EXP insertion, respectively. Demographics of patients in the TLE and EXP groups are presented in Table 1. The duration of observation (mean ± standard deviation) for the TLE and EXP groups were 20.5 ± 4.6 and 21.9 ± 3.9 months, respectively.

**Table 1:** Patient characteristics before filtration surgery

	TLE group (N = 48)	EXP group (N = 32)
Gender（male/female）	25/23	19/13
Age (Mean ± SD)	61.1 ± 10.0	71.9 ± 8.7
Type of glaucoma		
Primary open-angle glaucoma	33 (69%)	19 (59%)
Uveitic glaucoma	6 (13%)	0
Exfoliation glaucoma	4 (8%)	6 (19%)
Steroid-induced glaucoma	1 (2%)	2 (6%)
Pigmentary glaucoma	1 (2%)	2 (6%)
Neovascular glaucoma	1 (2%)	1 (3%)
Primary angle-closure glaucoma	1 (2%)	0
Traumatic glaucoma	1 (2%)	0
Undetermined	0	2 (6%)
ERM staging		
None	34	22
Stage 1	12	6
Stage 2	2	2
Stage 3	0	0
Stage 4	0	2

 Among the 34 eyes without preexisting ERMs before surgery in the TLE group, one (case 1) (3%) developed postoperative ERM, while out of the 14 eyes with preexisting ERMs, 1 (case 2) (7%) demonstrated progression. Out of the 22 eyes without preexisting ERMs before surgery in the EXP group, 1 (case 3) (5%) developed a postoperative ERM, and among the 10 eyes with preexisting ERM, one (case 4) (10%) demonstrated regression.

Case reports

Case 1

**Figure 2:**
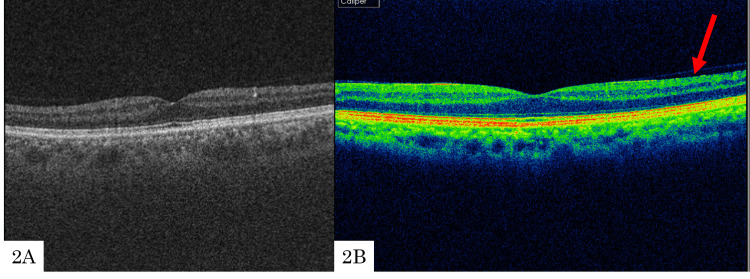
Optical coherence tomography preoperatively and at three months postoperatively Preoperatively, there was no evidence of an ERM (2A), but at the three-month follow-up, a stage 1 ERM was observed (2B).

A 58-year-old man with primary open-angle glaucoma (POAG) in the left eye underwent TLE. Preoperatively, there was no evidence of an ERM (Figure 2A), but at the three-month follow-up, a stage 1 ERM was observed (Figure 2B). His preoperative best corrected visual acuity (BCVA) (logMAR) in the right and left eyes was -0.18 and 0.30, respectively. Intraocular pressures (IOPs) in the right and left eyes were 15 and 17 mmHg, respectively. Axial lengths of the right and left eyes were 24.94 and 25.02 mm, respectively. The patient had no history of ocular surgery, and there was no history of retinal or inflammatory diseases. Upon postoperative ERM detection, posterior vitreous detachment (PVD) had not occurred. The mean deviation (MD), as measured by the Humphrey visual field analyzer (Carl Zeiss Inc., Dublin, CA; Swedish Interactive Threshold Algorithm (SITA)-standard, 10-2 program), was −32.01 dB in the left eye. Postoperatively, the IOP was 8-12 mmHg without additional procedures.

Case 2

**Figure 3:**
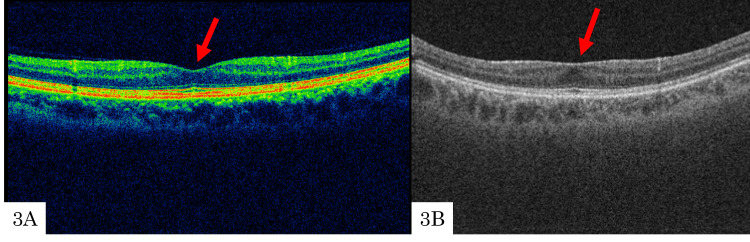
Optical coherence tomography preoperatively and at six months postoperatively Preoperatively, an ERM was classified as stage 1 (3A); at the six-month follow-up, the ERM progressed to stage 2 (3B).

A 49-year-old man with POAG in the right eye underwent TLE. Preoperatively, an ERM was classified as stage 1 (Figure 3A); at the six-month follow-up, the ERM progressed to stage 2 (Figure 3B). His preoperative BCVA in the right and left eyes was 0.046 and 0.52, respectively. IOPs in the right and left eyes were 15 and 13 mmHg, respectively. Axial lengths of the right and left eyes were 27.58 and 27.93 mm, respectively. The patient had previously undergone cataract surgery in the right eye two years before TLE. Fundus examination only showed an ERM and no other retinal or inflammatory diseases. Prior to surgery, PVD had not occurred, but by the time the stage 2 ERM was detected at the six-month follow-up, PVD was completed. The MD (10-2 program) measured −20.70 dB in the right eye. Postoperatively, the IOP ranged from 13 to 18 mmHg. Needling was performed one month after surgery, and filtration bleb revision was carried out at seven months postoperatively. Mitomycin C was not used during needling, but it was employed during filtration bleb revision. Following needle revision, the IOP ranged from 13 to 19 mmHg. After filtration bleb revision, the IOP remained in the range of 4-9 mmHg for one month and stabilized in the 10-mmHg range thereafter.

Case 3

**Figure 4:**
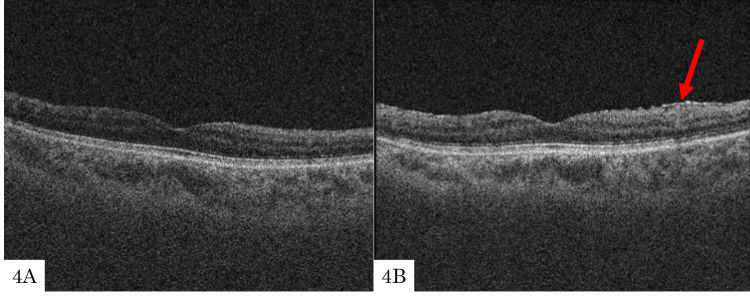
Optical coherence tomography preoperatively and at 15 months postoperatively Preoperatively, no ERMs were detected (4A), but at the 15-month follow-up, stage 1 ERM was observed (4B).

A 59-year-old woman with POAG in the right eye underwent EXP insertion. Preoperatively, no ERMs were detected (Figure 4A), but at the 15-month follow-up, stage 1 ERM was observed (Figure 4B). Her preoperative BCVA in her right and left eyes was 0 and -0.18, respectively. IOPs in the right and left eyes were 21 and 17 mmHg, respectively. Axial lengths of the right and left eyes were 27.96 and 27.77 mm, respectively. The patient had undergone cataract surgery in the right eye seven years prior to EXP insertion surgery. Preoperative fundus examination revealed no abnormal findings in the retina. The patient did not have a history of inflammatory diseases. Prior to EXP insertion, PVD had already occurred. The MD (10-2 program) measured −10.28 dB in the right eye. Postoperatively, the IOP ranged from 8 to 16 mmHg. No additional procedures were performed during the follow-up period.

Case 4

**Figure 5:**
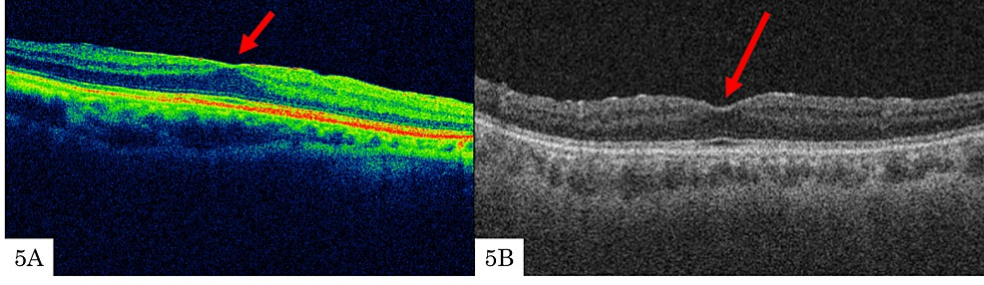
Optical coherence tomography preoperatively and at 12 months postoperatively Preoperatively, a stage 2 ERM was observed (5A), but at the 12-month follow-up, it regressed to stage 1 (5B).

A 72-year-old woman with steroid-induced glaucoma in the right eye underwent EXP insertion. She was concurrently receiving systemic steroid treatment for idiopathic thrombocytopenic purpura. Preoperatively, a stage 2 ERM was observed (Figure 5A), but at the 12-month follow-up, it regressed to stage 1 (Fig. 5B). Her preoperative BCVA in the right and left eyes was 0.046 and 0.52, respectively. IOPs in the right and left eyes were 25 and 12 mmHg, respectively. The axial lengths were not measured. Twelve years prior to EXP insertion, the patient had undergone combined trabeculotomy and cataract surgery in the right eye. She was undergoing treatment for diabetes mellitus, but diabetic retinopathy, or other retinal diseases, except for ERM, were not noted. She also had no history of inflammatory diseases. Prior to EXP insertion, PVD had already occurred. The MD (10-2 program) measured −22.76 dB in the right eye. Postoperatively, the intraocular pressure ranged from 11 to 16 mmHg. No additional procedures were performed during the follow-up period.

## Discussion

In this study, a new ERM following TLE was observed in one eye (3%), and ERM progression was noted in one eye (7%). Additionally, after EXP insertion, a new ERM developed in one eye (5%), and ERM regression was observed in one eye (10%).

There are reports on the impact of filtration surgery on the occurrence of both new and existing ERM. Vieira et al. reported that after TLE for POAG and exfoliation glaucoma (average age, 64.9 years), ERM developed in 18.8% of cases during an average follow-up of 27.5 months [10]. Loiudice et al. noted a 33% incidence rate of ERMs after EXP insertion for POAG (average age, 71.1 years) within six months, which was significantly higher than the 15% rate in the control group [11]. Potent inflammation following filtration surgery and forces exerted on the retina-vitreous interface due to rapid postoperative IOP fluctuations may potentially induce PVD and ERM formation [10,11]. TLE could also affect both the development of new ERMs and the progression of existing ERMs [10]. Furthermore, even in cases wherein surgery is not performed, Shin et al. reported a correlation between large IOP fluctuations and ERM development in patients with glaucoma [13].

In this study, cases 1 and 3 did not have ERMs preoperatively but developed ERMs postoperatively. Mitchell et al. reported that the prevalence of ERM was 1.9% in individuals under 60 years old, 7.2% in those in their 60s, 11.6% in their 70s, and 9.3% in those aged 80 years or older [14]. Both cases 1 and 3 underwent filtration surgery in their 50s, placing them in an age group with a relatively low ERM prevalence. However, postoperative ERMs developed in both cases. Considering the findings of previous studies [10,11,13], it is possible that inflammation and IOP fluctuations following TLE or EXP insertion may have influenced ERM formation. In case 2, the ERM was stage 1 preoperatively but progressed to stage 2 after TLE. TLE has been suggested to potentially influence both the development of new ERMs and the progression of existing ERMs [10]. In this case, TLE may have possibly contributed to the progression of ERM. Byon et al. reported that vitreoretinal attachment plays a role in the progression of idiopathic ERMs [15]. They found that eyes with vitreoretinal attachment showed a progression rate of 40% for idiopathic ERMs, whereas eyes with PVD exhibited only a 3.8% progression rate. In this case, PVD was not yet complete at the time of TLE, so apart from the impact of TLE, the vitreoretinal attachment should also be considered as having played a role in the progression of ERM. In case 4, the preoperative stage 2 ERM regressed to stage 1. Wetzel et al. reported cases wherein ERMs regressed following optic nerve atrophy, suggesting the possibility of the involvement of retinal nerve fiber layer atrophy and glial cell contraction [16]. In this case, changes in the retinal nerve fiber layer may have influenced the ERM and macular shape. Regarding ERM regression, Fraser-Bell et al. reported that during a five-year follow-up period, 28.6% of existing ERMs progressed, 38.8% remained stable, and 38.8% regressed [5]. Fong et al. observed that after cataract surgery in those with idiopathic ERMs, 43%, 32%, and 24% progressed, remained stable, and regressed, respectively [17]. These reports on the natural course of ERMs and ERM regression after cataract surgery suggest that factors other than EXP insertion may influence the grade of ERM. Compared with previously published studies that have evaluated ERM development following filtration surgery using OCT [10,11], this study revealed a lower incidence of postoperative ERMs. The prevalence of ERM varies across ethnicities and countries as assessed through fundus photography: 7% and 8.9% in Australia, 18.7% in the United States, 7.6%, 7.9%, and 12.1% in Singapore, 2.2% and 7.6% in China, 4.0% in Japan, and 2.9% in Korea [1]. These findings suggest the possibility of racial differences influencing study outcomes. Furthermore, postoperative inflammation and IOP fluctuations after filtration surgery can impact ERM development [10]. Therefore, differences in intraoperative and postoperative IOP fluctuations and variations in postoperative topical medications may differ from previous reports, potentially contributing to the disparity in the incidence of postoperative ERMs observed in this study.

There are two limitations to this study. First, since a control group was not included, it was not possible to compare ERM changes that were unrelated to surgery. Second, a history of intraocular surgery before filtration surgery may have influenced ERM development. This study focused on patients who had not undergone intraocular surgery within one year before filtration surgery; cases 2, 3, and 4 had a history of surgery more than a year before filtration surgery. The previous surgeries may have impacted ERM development during the long-term postoperative period. Hence, it cannot be ruled out that factors other than filtration surgery might have been involved in these cases.

## Conclusions

Glaucoma filtration surgery was suggested to be implicated in the development of new ERMs and the progression of preexisting ERMs. A preexisting ERM in one case regressed after filtration surgery. During follow-up and including the postfiltration surgery period, it is essential to assess for any changes in the status of retinal diseases.
